# Trends in Laboratory Evaluation and Risk Factors for Transfusion Therapy in Pediatric Epistaxis

**DOI:** 10.1016/j.acepjo.2026.100334

**Published:** 2026-02-12

**Authors:** Andrew Shieh, Angela C. Weyand, Lauren A. Bohm, James A. Cranford, Sarah E. Tomlinson

**Affiliations:** 1 Department of Emergency Medicine, University of Michigan, Ann Arbor, Michigan, USA; 2 Department of Pediatrics, Division of Hematology and Oncology, University of Michigan, Ann Arbor, Michigan, USA; 3 Department of Otolaryngology-Head and Neck Surgery, University of Michigan, Ann Arbor, Michigan, USA; 4 Department of Pediatrics, University of Michigan, Ann Arbor, Michigan, USA

**Keywords:** epistaxis, nosebleeds, pediatrics, emergency department, transfusion therapy, hematology

## Abstract

**Objectives:**

This study examines practices in laboratory evaluation and transfusion therapy for pediatric epistaxis in the emergency department (ED).

**Methods:**

We conducted a single-center retrospective study of children (<21 years) evaluated in the ED for epistaxis from 2013 to 2023. Extracted data included demographics, medical history, laboratory evaluation, and treatments. Laboratory testing included complete blood count (CBC) and coagulation tests. We performed descriptive analyses to describe factors associated with laboratory testing and transfusion therapy.

**Results:**

Of 944 children (median age 8.8 years; interquartile range, 4.2-14.2 years), 296 (31.4%) underwent laboratory testing. Among recipients, 119 (40.2%) received a CBC alone and 174 (58.7%) received both CBC and coagulation tests. Factors associated with laboratory testing included older age, White race, use of antiplatelet medications, recent nasal procedures, and sinonasal vascular malformations. Transfusions were administered to 46 children (4.9%), including blood (*n* = 9, 19.6%), platelets (*n* = 10, 21.7%), factor replacement (*n* = 11, 23.9%), and multiple products (*n* = 16, 34.8%). Factors associated with undergoing both laboratory evaluation and transfusion included prolonged epistaxis, active bleeding at presentation, recurrent epistaxis within 24 hours, a known bleeding disorder, an existing oncological condition, anticoagulant use, a prior history of epistaxis, and nasal interventions performed in the ED.

**Conclusion:**

Laboratory evaluation and transfusions are infrequently performed in pediatric epistaxis. However, prolonged or recurrent bleeding, underlying bleeding disorders, existing oncological conditions, anticoagulant use, and nasal interventions performed to control bleeding are associated with an increased likelihood of laboratory testing and transfusion therapy in the ED.

**The Bottom Line**

Epistaxis is common in children, although most studies focus on ambulatory settings. Many patients present to the emergency department (ED) for potential laboratory evaluation and transfusions. In this single-center retrospective study, we observed patterns in laboratory testing and transfusion practices for pediatric epistaxis. Factors associated with transfusions included prolonged or recurrent bleeding, underlying bleeding disorders, existing oncological conditions, anticoagulant use, prior epistaxis, and nasal interventions performed in the ED. These findings suggest that laboratory and transfusion practices are guided primarily by patient-specific risk factors.

## 1 Introduction

### 1.1 Background

Epistaxis is a common self-limited problem among children, occurring in 30% of those less than 5 years of age, 56% of those 6-10 years, and 64% of children more than 11 years old.^1^, ^2^, ^3^, ^4^ Previous studies have evaluated the causes and treatments of epistaxis in children, primarily occurring in ambulatory settings where patients are referred for further assessment by specialists.^3^, ^4^, ^5^, ^6^, ^7^, ^8^, ^9^, ^10^, ^11^ Nevertheless, it is also common for patients to arrive at the emergency department (ED) because of concerns regarding urgent medical interventions needed during or immediately following a bleeding episode. The number of studies focused on pediatric cases in the ED is limited.^1^^,^^12^^,^^13^

Most cases of pediatric epistaxis are spontaneous, do not require urgent laboratory evaluation, and resolve entirely with conservative treatment.^1^^,^^6^^,^^14^ The management of epistaxis is highly variable, ranging from the application of direct pressure to the nares to more invasive approaches such as cautery, packing, or embolization.^15^ The Clinical Practice Guidelines published by the American Academy of Otolaryngology - Head, and Neck Surgery in 2020 outlined management options for epistaxis and emphasized the significance of direct pressure, followed by topical vasoconstrictors, packing, and cautery in a stepwise approach.^16^ However, current guidelines do not specify patterns of laboratory testing, and the optimal approach for guiding transfusion therapy remains unclear.

### 1.2 Importance

The etiology of epistaxis varies with the patient’s age and may result from local sinonasal or systemic causes, such as medication use and underlying coagulopathy.^3^^,^^6^^,^^12^^,^^17^ Identifying the cause is crucial, as children with specific risk factors require urgent examination.^12^^,^^15^ There is no agreement on the best approach to the standard work-up for pediatric epistaxis in an urgent context, and a comprehensive understanding would have significant implications for the appropriate use of emergency services.

### 1.3 Goals of This Investigation

Our aim was to examine the incidence, demographics, comorbidities, and treatment of children presenting with epistaxis in the ED. We also sought to characterize current trends in laboratory evaluation and to identify risk factors associated with transfusion therapy in this population.

## 2 Methods

### 2.1 Study Design

We conducted a retrospective analysis of children presenting to the ED with epistaxis. Patients were initially stratified into 2 groups based on receipt of laboratory evaluation, which included complete blood count (CBC) and/or coagulation assessments (international normalized ratio and partial thromboplastin time). Patients were also classified into 2 groups according to receipt of transfusion therapy, including blood, platelet, or factor replacement transfusions. We performed a separate analysis excluding patients with known elevated risk of bleeding, including those with bleeding disorders, oncological conditions, use of antiplatelet or anticoagulant medications, recent nasal procedures, and sinonasal vascular malformations.

### 2.2 Setting

This study was conducted at a single academic, quaternary-care pediatric hospital in the Midwest. Our ED provides care for approximately 45,000 children and young adults annually. There is no standardized algorithm for the assessment and management of epistaxis at our institution. At our center, transfusions may be administered in the ED or necessitate hospital admission, contingent on the preferences of the ED physician and specialists.

### 2.3 Selection of Participants

We queried the electronic medical record at our institution utilizing International Classification of Diseases (ICD) codes for “epistaxis” (ICD-9 code 784.7 and ICD-10 code R04.0) to identify all visits for children (<21 years) with a primary diagnosis of epistaxis in the ED from January 1, 2013, to December 31, 2023. We reviewed all cases and included all patients who were primarily evaluated for and diagnosed with epistaxis during the ED visit. ED visits with incomplete data were excluded. We randomly selected 1 visit for patients with multiple encounters for epistaxis.

### 2.4 Measurements

The data extracted from the electronic medical record included demographics, medical history, laboratory evaluations, and treatments administered in the ED. All data were documented in REDCap (Vanderbilt University).^18^^,^^19^ We verified the extracted data for accuracy to ensure patients were correctly categorized based on the receipt of laboratory testing and that transfusions were ordered within the ED setting. The first and fourth authors reviewed all data for completeness and evaluated interrater reliability through a 30% random audit of the data (Table S1).

### 2.5 Outcomes

The primary outcome was the receipt of a laboratory evaluation. The secondary outcome was the receipt of transfusion therapy.

### 2.6 Data Analysis

Categoric data were presented in frequencies and percentages. Age was described as median, range, and categoric data. The Pearson *χ*^2^, Fisher’s exact, and Mann-Whitney U tests compared demographics, risk factors, clinical characteristics, and treatments administered between groups based on receipt of laboratory evaluation. Additional Pearson *χ*^2^, Fisher’s exact, and Mann-Whitney U tests analyzed associations between demographics and clinical variables with receipt of transfusions. In a separate analysis, we compared characteristics between groups, stratified by receipt of laboratory evaluation, among excluded patients who had ED revisits (Table S2). A 2-tailed *P* value of < .05 was deemed statistically significant for all associations. All analyses were performed using SPSS Statistics Version 28 (IBM Corporation).

### 2.7 Ethics Approval

The study was approved by the University of Michigan institutional review board #00205713. Informed consent was waived due to the retrospective nature and the use of de-identified data.

## 3 Results

### 3.1 Patient Characteristics

During the study period, a total of 337,038 ED encounters were eligible for inclusion. We identified 1107 (0.33%) visits with a diagnosis of epistaxis. We excluded 96 visits due to incomplete data and 67 revisits to the ED by 50 patients. Our analysis included 944 visits representing unique patients, of which 296 (31.4%) underwent laboratory evaluation. Among recipients, 119 (40.2%) had only a CBC, 4 (1.4%) had only coagulation tests, and 174 (58.7%) underwent both tests. *χ*^2^ tests comparing demographics, medical history, clinical characteristics, and treatments administered are described in detail in Table 1. The median age of the cohort was 8.8 years (interquartile range, 4.2-14.2), and 528 (55.9%) of the patients were male.

**Table 1:** Comparison of all patients by receipt of laboratory evaluation.

	All (*N* = 944)	No laboratory evaluation (*n* = 648)	Received laboratory evaluation (*n* = 296)	*P* value
Age, y				<.001
Median (range)	8.81 (0.02-20.94)	7.37 (0.02-20.94)	12.28 (0.42-20.56)	
Age, categoric				<.001
Age <10 y	531 (56)	416 (64)	115 (39)	
Age ≥10 y	413 (44)	232 (36)	181 (61)	
Sex				.105
Male	528 (56)	374 (58)	154 (52)	
Female	416 (44)	274 (42)	142 (48)	
Race				.002
White/Caucasian	566 (60)	370 (57)	196 (66)	
Black/African American	186 (20)	147 (23)	39 (13)	
Asian	74 (8)	56 (8)	18 (6)	
Other	118 (12)	75 (12)	43 (15)	
Insurance				.292
Government	377 (40)	262 (40)	115 (39)	
Private	543 (58)	373 (58)	170 (57)	
Self-pay	24 (2)	13 (2)	11 (4)	
Time of arrival				.514
7pm-7am	349 (37)	235 (36)	114 (38)	
7am-7pm	595 (63)	413 (64)	182 (62)	
Duration of nosebleed				<.001
<30 min	614 (65)	481 (74)	133 (45)	
≥30 min	330 (35)	167 (26)	163 (55)	
Frequency of epistaxis				<.001
1 nosebleed within 24 h	634 (67)	477 (74)	157 (53)	
>1 nosebleed within 24 h	310 (33)	171 (26)	139 (47)	
Active bleeding in ED	139 (15)	52 (8)	87 (29)	<.001
Previous history of epistaxis	327 (35)	161 (25)	166 (56)	<.001
Medical history				
None	327 (35)	211 (33)	116 (40)	.055
Upper respiratory illness	279 (30)	208 (32)	71 (24)	.011
Trauma	229 (24)	211 (33)	18 (6)	<.001
Bleeding disorders	64 (7)	12 (2)	52 (18)	<.001
Oncological conditions	23 (2)	1 (0.2)	22 (7)	<.001
Antiplatelet medication use	26 (3)	7 (1)	19 (6)	<.001
Anticoagulant medication use	12 (1)	3 (1)	9 (3)	.002
Nasal procedure within past 30 days	35 (4)	16 (3)	19 (6)	.005
Sinonasal vascular malformations	5 (0.5)	1 (0.2)	4 (1)	.036
Nasal intervention in ED				
Intranasal medication	191 (20)	98 (15)	93 (31)	<.001
Packing	44 (5)	8 (1)	36 (12)	<.001
Silver nitrate cauterization	30 (3)	14 (2)	16 (5)	.008

Data shown as *n* (%) unless otherwise indicated.

All categoric characteristics were analyzed using the *χ*^2^ test, except for anticoagulant medication use and sinonasal vascular malformations, which were analyzed using Fisher’s exact test. Median age was compared using the Mann-Whitney U test.

ED, emergency department.

### 3.2 Primary Outcome

Laboratory evaluation was associated with older age (median 12.3 compared with 7.4 years, *P < .001*) and race (*P = .002*). Clinical characteristics associated with laboratory evaluation included prolonged epistaxis, active bleeding at presentation, recurrent epistaxis within 24 hours, and nasal interventions performed in the ED. Medical conditions associated with laboratory evaluation included the presence of bleeding disorders, presence of oncological conditions, use of antiplatelet medications, use of anticoagulant medications, recent nasal procedures, sinonasal vascular malformations, and a previous history of epistaxis. Von Willebrand disease was the most common previously known bleeding disorder, and leukemia was the most frequently observed oncologic condition (Table 2). Among 794 patients without previously identified bleeding risk, older age, prolonged epistaxis, active bleeding, recurrent bleeding, a prior history of epistaxis, and nasal interventions performed in the ED were associated with laboratory evaluation (Table 3).

**Table 2:** Types of disorders, procedures, malformations, and conditions requiring antiplatelet or anticoagulant therapy.

Bleeding disorder	Frequency (*n* = 64)
Von Willebrand disease	22 (34)
Immune thrombocytopenia	19 (30)
Other thrombocytopenia	12 (19)
Hemophilia A	4 (6)
Other	7 (11)
Oncological condition	Frequency (*n* = 23)
Leukemia	10 (44)
Lymphoma	6 (26)
Other	7 (30)
Nasal procedure within the past 30 d	Frequency (*n* = 35)
Adenoidectomy	16 (46)
Cauterization	5 (14)
Other	14 (40)
Condition requiring antiplatelet medication	Frequency (*n* = 26)
Cardiac disorder	10 (39)
Postoperative pain	6 (23)
Prior thrombosis	4 (15)
Neurologic disorder	2 (8)
Other	4 (15)
Condition requiring anticoagulant medication	Frequency (*n* = 12)
Cardiac disorder	7 (58)
Prior thrombosis	5 (42)
Sinonasal vascular malformations	Frequency (*n* = 5)
Arteriovenous malformation	3 (60)
Angiofibroma	2 (40)

Data shown as *n* (%).

**Table 3:** Comparison of patients without identified bleeding risk by receipt of laboratory evaluation.

	All (*N* = 794)	No laboratory evaluation (*n* = 610)	Received laboratory evaluation (*n* = 184)	*P* value
Age, y				<.001
Median (range)	8.28 (0.02-20.94)	7.44 (0.02-20.94)	11.77 (0.42-20.56)	
Age, categoric				<.001
Age <10 y	467 (59)	390 (64)	77 (42)	
Age ≥10 y	327 (41)	220 (36)	107 (58)	
Sex				.122
Male	445 (56)	351 (58)	94 (51)	
Female	349 (44)	259 (42)	90 (49)	
Race				.078
White/Caucasian	468 (59)	346 (57)	122 (66)	
Black/African American	166 (21)	139 (23)	27 (15)	
Asian	68 (8)	53 (8)	15 (8)	
Other	92 (12)	72 (12)	20 (11)	
Insurance				.235
Government	310 (39)	242 (40)	68 (37)	
Private	463 (58)	355 (58)	108 (59)	
Self-pay	21 (3)	13 (2)	8 (4)	
Time of arrival				.420
7pm-7am	499 (63)	388 (64)	111 (60)	
7am-7pm	295 (37)	222 (36)	73 (40)	
Duration of nosebleed				<.001
<30 min	563 (71)	457 (75)	106 (58)	
≥30 min	231 (29)	153 (25)	78 (42)	
Frequency of epistaxis				<.001
1 nosebleed within 24 h	551 (69)	457 (75)	94 (51)	
>1 nosebleed within 24 h	243 (31)	153 (25)	90 (49)	
Active bleeding in ED	75 (9)	44 (7)	31 (17)	<.001
Previous history of epistaxis	243 (31)	150 (25)	93 (50)	<.001
Medical history				
None	327 (41)	211 (35)	116 (63)	<.001
Upper respiratory illness	258 (32)	203 (33)	55 (30)	.390
Trauma	220 (28)	207 (34)	13 (7)	<.001
Nasal intervention in ED				
Intranasal medication	125 (16)	84 (14)	41 (22)	.005
Packing	19 (2)	6 (1)	13 (7)	<.001
Silver nitrate cauterization	21 (3)	12 (2)	9 (5)	.038

Data shown as n (%) unless otherwise indicated.

All categoric characteristics were analyzed using the *χ*^2^ test, except for packing and silver nitrate cauterization, which were analyzed using Fisher’s exact test. Median age was compared using the Mann-Whitney U test.

ED, emergency department.

### 3.3 Secondary Outcome

Of 46 (4.9%) children who received transfusions, 9 (19.6%) received blood, 10 (21.7%) received platelets, 11 (23.9%) received factor replacement, and 16 (34.8%) received multiple products. Children who received transfusion therapy had lower hemoglobin (median 8.3 compared with 12.6 g/dL, *P < .001*) and lower platelet (median 23.5 compared with 247 K/*μ*L, *P < .001*) values. International normalized ratio (median 1.2 compared with 1.1, *P = .959*) and partial thromboplastin time (median 28.4 compared with 26.8 seconds, *P = .209*) did not differ significantly between transfusion and nontransfusion groups. Factors associated with transfusion therapy included prolonged or recurrent epistaxis, active bleeding on presentation, a history of prior epistaxis, and nasal interventions performed in the ED (Table 4). Transfusion therapy was associated with underlying medical conditions, including bleeding disorders, oncologic diagnoses, and anticoagulant use. Among all children who received transfusions, 19 (41.3%) were managed in the ED and discharged home. In patients without a pre-existing bleeding risk, 2 (0.3%) required transfusion, including 1 with a new oncologic diagnosis and 1 with a newly identified bleeding disorder. Of the 67 excluded patients who returned to the ED, transfusion was necessary in 2 (3.0%) cases—1 involving an oncologic condition and the other a known bleeding disorder.

**Table 4:** Comparison of all patients by receipt of transfusion therapy.

	No transfusion therapy (*n* = 898)	Received transfusion therapy (*n* = 46)	*P* value
Age, y			.363
Median (range)	8.81 (0.02-20.94)	8.92 (0.63-20.13)	
Age, categoric			.568
Age <10 y	507 (57)	24 (52)	
Age ≥10 y	391 (43)	22 (48)	
Sex			.319
Male	499 (56)	29 (63)	
Female	399 (44)	17 (37)	
Race			.207
White/Caucasian	539 (60)	27 (59)	
Black/African American	179 (20)	7 (15)	
Asian	72 (8)	2 (4)	
Other	108 (12)	10 (22)	
Insurance			.603
Government	357 (40)	20 (44)	
Private	519 (58)	24 (52)	
Self-pay	22 (2)	2 (4)	
Time of arrival			.756
7pm-7am	331 (37)	18 (39)	
7am-7pm	567 (63)	28 (61)	
Duration of nosebleed			<.001
<30 min	605 (67)	9 (20)	
≥30 min	293 (33)	37 (80)	
Frequency of epistaxis			.011
1 nosebleed within 24 h	611 (68)	23 (50)	
>1 nosebleeds within 24 h	287 (32)	23 (50)	
Active bleeding in ED	113 (13)	26 (57)	<.001
Previous history of epistaxis	295 (33)	32 (70)	<.001
Medical history			
None	325 (36)	2 (4)	<.001
Upper respiratory illness	272 (30)	7 (15)	.029
Trauma	227 (25)	2 (4)	.001
Bleeding disorders	45 (5)	19 (41)	<.001
Oncological conditions	4 (0.4)	19 (41)	<.001
Antiplatelet medication use	23 (3)	3 (7)	.129
Anticoagulant medication use	7 (1)	5 (11)	<.001
Nasal procedure within past 30 d	32 (4)	3 (7)	.240
Sinonasal vascular malformations	4 (0.4)	1 (2)	.221
Nasal intervention in ED			
Intranasal medication	167 (19)	24 (52)	<.001
Packing	34 (4)	10 (22)	<.001
Silver nitrate cauterization	28 (3)	2 (4)	.654
ED disposition			<.001
Discharge to home	866 (96)	19 (41)	
Admission to hospital	32 (4)	27 (59)	

Data shown as *n* (%) unless otherwise indicated.

All categoric characteristics were analyzed using the *χ*^2^ test, except for bleeding disorders, oncological conditions, antiplatelet and anticoagulant medication use, nasal procedures within the past 30 days, sinonasal vascular malformations, packing, silver nitrate cauterization, and ED disposition, which were analyzed using Fisher’s exact test. Median age was compared using the Mann-Whitney U test.

ED, emergency department.

## 4 Limitations

This study was conducted at a single academic children’s hospital ED, which restricts the applicability of the findings to nonacademic hospitals or EDs that do not regularly treat children. Due to the retrospective nature and dependence on ICD-9 and ICD-10 codes for identifying encounters, the data collected are contingent on the thoroughness of the coding within the electronic medical record. The exclusion of ED visits with incomplete data may have introduced selection bias, potentially leading to an underestimation of the true frequency of study outcomes. In particular, excluding patients with prolonged or active bleeding or a prior history of epistaxis omits individuals who are more likely to undergo laboratory evaluation and receive transfusions. Consequently, the observed frequencies of these interventions in the ED are likely underestimated. This selection bias may reduce the internal validity of the study and limit the generalizability of the findings to the broader pediatric population. A comprehensive assessment of additional clinical factors (such as joint pain, bruising, and fatigue) was not performed, as this information was not routinely documented. The origin of the bleeding, whether from the anterior septum or posterior sources, could not be confirmed. Only a small number of patients had sinonasal vascular malformations, were receiving anticoagulant or antiplatelet therapy, or had a documented oncological history. The low prevalence of these risk factors rendered the data insufficient for stable parameter estimation, thereby precluding the use of a multivariable model without an unacceptable risk of overfitting. In the absence of larger studies, identifying definitive trends in laboratory evaluation is challenging, and the results should be interpreted with caution. Other specific reasons for laboratory evaluation, such as caregiver or pediatrician concerns and variations in ED provider practice, were not captured. It is also possible that patients sought treatment at prehospital clinics before arriving, which may have influenced our providers’ decisions regarding laboratory testing. A greater number of patients received transfusions compared with those who were treated with packing or cautery, underscoring the variability in management, as some patients may have received unnecessary transfusions. Finally, our descriptive analysis explores correlations between patient characteristics and the use of laboratory evaluation or transfusions, without suggesting causality. Nevertheless, our findings provide valuable insights into the current gaps in the literature regarding trends in laboratory testing and transfusion practices in pediatric epistaxis.

## 5 Discussion

This study provides insight into which children presenting with epistaxis underwent laboratory evaluation and received transfusion therapy in a single pediatric ED. Demographic factors, including older age and White race, as well as clinical features such as antiplatelet use, recent nasal procedures, and sinonasal vascular malformations, were associated with laboratory testing but not with transfusions. In contrast, children with prolonged or recurrent bleeding, underlying bleeding disorders, oncological conditions, anticoagulant use, prior epistaxis, or nasal interventions performed in the ED were more likely to receive transfusions and should be prioritized for urgent laboratory evaluation.

The exact incidence of pediatric epistaxis remains uncertain, as most cases resolve spontaneously and do not require medical intervention. A prior study reported that 0.46% of all ED visits, including both pediatric and adult patients, from 1992 to 2001, were attributed to epistaxis.^20^ Our observed incidence rate over an 11-year period was 0.33%. This slightly lower rate likely reflects our exclusive focus on patients under 21 years of age and the availability of comprehensive ambulatory services at our institution, which facilitate direct communication between patients and their primary care providers. The racial distribution of our cohort, with 60% White patients, mirrors the local population, and the male predominance aligns with prior reports of pediatric epistaxis.^1^, ^2^, ^3^, ^4^, ^5^, ^6^^,^^8^^,^^13^, ^14^, ^15^^,^^20^ The median age of our patients was 8.8 years, which closely corresponds to the mean age at presentation reported in both ambulatory and ED studies.^1^^,^^3^^,^^5^^,^^13^^,^^14^ Consistent with existing literature, the majority of epistaxis cases in our cohort were brief and self-resolving.^6^^,^^8^^,^^14^

Previous studies have shown that the primary causes of pediatric epistaxis are local, including nasal trauma, viral infections, and idiopathic etiologies.^2^^,^^6^ In our study, >60% of patients had a comorbidity related to epistaxis, with local factors more prevalent than systemic causes. Our cohort included 7% of patients with bleeding disorders and 2% with oncological conditions, exceeding previously reported rates of 4.5% for coagulation disorders and 0.2% for hematologic malignancies among children treated in the ED for epistaxis.^1^ This higher prevalence likely reflects the quaternary level of care provided at our facility. Consistent with prior studies, thrombocytopenia and von Willebrand disease were the most common bleeding disorders associated with pediatric epistaxis.^10^^,^^15^^,^^21^ Previous literature reported that acute hemorrhage complicating procedures accounted for 1.3% of admissions.^15^ In our cohort, 4% of cases were associated with recent procedures, although not all exhibited active bleeding. Active bleeding was observed in 15% of patients, and most cases did not require invasive interventions such as packing or cauterization, which aligns with existing literature.^1^^,^^6^^,^^12^^,^^13^

Our results indicated that White children were more likely to undergo laboratory testing. In a study of 372 admitted children, White patients comprised 39% of the cohort, suggesting they may present with more severe epistaxis warranting hospital admission.^21^ A large cross-sectional analysis also found that White children were more likely to undergo ligation and embolization procedures during hospitalizations, although this difference was not statistically significant.^15^ The presence of racial and ethnic disparities in the management of pediatric otolaryngologic conditions has been documented beyond epistaxis.^22^^,^^23^ Our findings complement previous literature, highlighting potential racial disparities in diagnostic assessment. Addressing these disparities through larger studies that better characterize laboratory testing patterns could improve patient care and optimize resource utilization.

At our institution, laboratory testing was frequently performed in children with a history of prior epistaxis, active bleeding, or recurrent epistaxis within 24 hours. However, past risk factors, such as a personal history of bleeding, have been shown to be poor predictors of underlying coagulopathy.^5^ Laboratory evaluations in children with recurrent nosebleeds in ambulatory settings have revealed few cases of thrombocytopenia.^8^^,^^10^^,^^11^ Other studies report a coagulopathy prevalence ranging from 6% to 30% among children, although it is unclear whether these patients required factor replacement or blood transfusions.^5^^,^^8^^,^^10^^,^^13^^,^^24^ Similarly, Patel et al^3^ found that 21% of 131 otherwise healthy children presenting with epistaxis at a tertiary care clinic had anemia, although none required transfusions.^3^ In our cohort, among children without known bleeding disorders, 8 children presenting with bruising were diagnosed with immune thrombocytopenia, highlighting the importance of thorough physical examinations. One patient presenting with leg pain was subsequently diagnosed with leukemia. Additionally, 2 patients with a history of epistaxis were later diagnosed with von Willebrand disease in the outpatient setting; only 1 had coagulation testing performed in the ED, which was normal. Consistent with the findings of Hadar et al,^13^ our results indicate that most children without previously identified bleeding risk do not undergo routine laboratory testing in the ED and that transfusion therapy in this population is rare.

Our study indicated that children with systemic causes, such as bleeding disorders, oncological conditions, or anticoagulant therapy, or who underwent nasal interventions in the ED, were frequently subjected to both laboratory evaluation and transfusion therapy. Similarly, Katsanis et al^25^ reported anemia in 33% of 36 children presenting with epistaxis, with those experiencing severe bleeding more likely to require cautery, although none had a coagulopathy necessitating factor replacement transfusions. Shay et al^1^ found that coagulation disorders and malignancies were not associated with the use of packing or cautery. Our results extend this literature, demonstrating that transfusions were more common among patients with bleeding disorders and cancer-related conditions. Analysis of excluded ED revisits identified only 2 cases of bleeding that required transfusion, suggesting that the aggressive transfusion strategy used for patients in the main cohort was generally appropriate. The transfusion rate in our cohort was 5%, closely aligning with the 4% rate reported in a prior study of 231 children presenting to the ED.^13^ In contrast, an analysis of >11,000 hospitalized children revealed a 24% transfusion rate, with those requiring packing more likely to receive transfusions than those undergoing aggressive surgical procedures.^15^ This substantial variation in transfusion rates across different settings highlights the absence of standardized criteria for transfusion in pediatric epistaxis. In a smaller subset, sinonasal vascular malformations were not associated with transfusion therapy in our study; further large-scale investigations are needed to examine children with structural abnormalities and validate these findings. Contrary to Baugh and Chang, who identified acute posthemorrhagic anemia following procedures as a common indication for ligation,^15^ our findings suggest that recent nasal procedures were not associated with transfusion therapy. Additional studies are warranted to establish specific laboratory thresholds for transfusion in children with postoperative anemia, thereby promoting timely management and optimized resource utilization in the ED.

In summary, we describe current practices for emergent laboratory testing in children presenting with epistaxis. Laboratory evaluation is frequently performed in patients with prolonged or recurrent bleeding, underlying bleeding disorders, oncological conditions, anticoagulant use, a history of prior epistaxis, or those requiring nasal interventions in the ED, to assess the potential need for transfusion therapy. Our findings suggest that emergency physicians can optimize resource utilization by prioritizing laboratory testing for children with these risk factors, thereby more effectively determining which patients may require transfusions.

## Author Contributions

AS and SET developed the research question and obtained institutional approval. SET supervised the research. AS retrieved and collected the data. AS and JAC performed the data analysis and interpretation. AS prepared the manuscript. All authors revised the manuscript and approved the version as submitted.

## Funding and Support

By *JACEP* *Open* policy, all authors are required to disclose any and all commercial, financial, and other relationships in any way related to the subject of this article as per ICMJE conflict of interest guidelines (see www.icmje.org). The authors have stated that no such relationships exist.

## Conflict of Interest

All authors have affirmed they have no conflicts of interest to declare.

## Supplemental material

Supplementaty Material
