# Supplementary material for: Trends in Laboratory Evaluation and Risk Factors for Transfusion Therapy in Pediatric Epistaxis
Source: J Am Coll Emerg Physicians Open. 2026 Feb 12;7(2):100334. doi: 10.1016/j.acepjo.2026.100334 (PMC12914786; doi:10.1016/j.acepjo.2026.100334)
Supplement: Supplementaty Material [file mmc1.docx]

**Supplementary Table S1. Interrater Reliability of Study Variables**

| **Variable** | **Result** |
| --- | --- |
| Age | ICC = 1.00 |
| Sex | Cohen’s κ = 1.00 |
| Race | Cohen’s κ = 1.00 |
| Insurance | Cohen’s κ = 1.00 |
| Time of Arrival | Cohen’s κ = 1.00 |
| Duration of Nosebleed | Cohen’s κ = 0.86 |
| Active Bleeding in ED | Cohen’s κ = 0.82 |
| Frequency of Epistaxis | Cohen’s κ = 0.83 |
| Medical History | Cohen’s κ = 0.93 |
| Nasal Intervention | Cohen’s κ = 1.00 |
| Laboratory Evaluation | Cohen’s κ = 1.00 |
| Transfusion Therapy | Cohen’s κ = 1.00 |

ICC= intraclass correlation coefficient.

**Supplementary Table S2. Comparison of Patients with ED Revisits by Receipt of Laboratory Evaluation**

|  | **All (N=67)** | **No Laboratory Evaluation (n=32)** | **Received Laboratory Evaluation (n=35)** | ***p* value** |
| --- | --- | --- | --- | --- |
| **Age, Years** | | | | *0.001* |
| Median (range) | 13.00 (1.35-20.35) | 10.32 (1.35-19.95) | 14.54 (2.37-20.35) |  |
| **Age, Categorical** | | | | *0.010* |
| Age <10 Years | 23 (34) | 16 (50) | 7 (20) |  |
| Age ≥10 Years | 44 (66) | 16 (50) | 28 (80) |  |
| **Sex** | | | | *0.361* |
| Male | 38 (57) | 20 (63) | 18 (51) |  |
| Female | 29 (43) | 12 (37) | 17 (49) |  |
| **Race** | | | | *0.308* |
| White/Caucasian | 32 (48) | 16 (50) | 16 (46) |  |
| Black/African American | 11 (16) | 6 (19) | 5 (14) |  |
| Asian | 2 (3) | 2 (6) | 0 (0) |  |
| Other | 22 (33) | 8 (25) | 14 (40) |  |
| **Insurance** | | | | *0.401* |
| Government | 32 (48) | 17 (53) | 15 (43) |  |
| Private | 35 (52) | 15 (47) | 20 (57) |  |
| Self-Pay | 0 (0) | 0 (0) | 0 (0) |  |
| **Time of Arrival** | | | | *0.612* |
| 7PM-7AM | 44 (66) | 22 (69) | 22 (63) |  |
| 7AM-7PM | 23 (34) | 10 (31) | 13 (37) |  |
| **Duration of Nosebleed** | | | | *<0.001* |
| <30 Minutes | 28 (42) | 21 (66) | 7 (20) |  |
| ≥30 Minutes | 39 (58) | 11 (34) | 28 (80) |  |
| **Frequency of Epistaxis** | | | | *0.273* |
| 1 Nosebleed Within 24 Hours | 33 (49) | 18 (56) | 15 (43) |  |
| >1 Nosebleed Within 24 Hours | 34 (51) | 14 (44) | 20 (57) |  |
| **Active Bleeding in ED** | 25 (37) | 4 (12) | 21 (60) | *<0.001* |
| **Previous History of Epistaxis** | 46 (69) | 19 (59) | 27 (77) | *0.117* |
| **Medical History** | | | |  |
| None | 19 (28) | 14 (44) | 5 (14) | *0.008* |
| Upper Respiratory Illness | 15 (22) | 9 (28) | 6 (17) | *0.281* |
| Trauma | 9 (13) | 4 (12) | 5 (14) | *1.000* |
| Bleeding Disorders | 17 (25) | 4 (12) | 13 (37) | *0.021* |
| Oncological Conditions | 5 (7) | 0 (0) | 5 (14) | *0.054* |
| Antiplatelet Medication Use | 4 (6) | 1 (3) | 3 (9) | *0.615* |
| Anticoagulant Medication Use | 3 (4) | 0 (0) | 3 (9) | *0.240* |
| Nasal Procedure Within Past 30 Days | 3 (4) | 1 (3) | 2 (6) | *1.000* |
| Sinonasal Vascular Malformations | 1 (1) | 1 (3) | 0 (0) | *0.478* |
| **Nasal Intervention in ED** | | | |  |
| Intranasal Medication | 30 (45) | 9 (28) | 21 (60) | *0.009* |
| Packing | 13 (19) | 2 (6) | 11 (31) | *0.009* |
| Silver Nitrate Cauterization | 4 (6) | 1 (3) | 3 (9) | *0.615* |

Data from 50 patients with 67 ED revisits are reported as n (%) unless specified otherwise.

All categorical characteristics were analyzed using the χ² test, except for trauma, oncological conditions, antiplatelet and anticoagulant medication use, nasal procedures within the past 30 days, sinonasal vascular malformations, and silver nitrate cauterization, which were analyzed using Fisher’s exact test. Median age was compared using the Mann-Whitney U test.

ED= emergency department.
